# Effect of amiloride, or amiloride plus hydrochlorothiazide, versus hydrochlorothiazide on glucose tolerance and blood pressure (PATHWAY-3): a parallel-group, double-blind randomised phase 4 trial

**DOI:** 10.1016/S2213-8587(15)00377-0

**Published:** 2016-02

**Authors:** Morris J Brown, Bryan Williams, Steve V Morant, David J Webb, Mark J Caulfield, J Kennedy Cruickshank, Ian Ford, Gordon McInnes, Peter Sever, Jackie Salsbury, Isla S Mackenzie, Sandosh Padmanabhan, Thomas M MacDonald

**Affiliations:** a Clinical Pharmacology Unit, Addenbrooke's Hospital, University of Cambridge, Cambridge, UK; b Institute of Cardiovascular Sciences, University College London, London, UK; c National Institute for Health Research, University College London Hospitals Biomedical Research Centre, London, UK; d Medicines Monitoring Unit, Medical Research Institute, University of Dundee, Dundee, Scotland, UK; e Clinical Pharmacology Unit, University of Edinburgh, Centre for Cardiovascular Science, Queen's Medical Research Institute, Edinburgh, Scotland, UK; f William Harvey Research Institute, Barts and the London School of Medicine and Dentistry, Queen Mary University of London, London, UK; g Cardiovascular Medicine & Diabetes, King's College London, London, UK; h Robertson Centre for Biostatistics, University of Glasgow, Glasgow, Scotland, UK; i BHF Glasgow Cardiovascular Research Centre, Institute of Cardiovascular and Medical Sciences, University of Glasgow, Glasgow, Scotland, UK; j International Centre for Circulatory Health, Imperial College London, London, UK

## Abstract

**Background:**

Potassium depletion by thiazide diuretics is associated with a rise in blood glucose. We assessed whether addition or substitution of a potassium-sparing diuretic, amiloride, to treatment with a thiazide can prevent glucose intolerance and improve blood pressure control.

**Methods:**

We did a parallel-group, randomised, double-blind trial in 11 secondary and two primary care sites in the UK. Eligible patients were aged 18–80 years; had clinic systolic blood pressure of 140 mm Hg or higher and home systolic blood pressure of 130 mmHg or higher on permitted background drugs of angiotensin-converting enzyme inhibitors, angiotensin-receptor blockers, β blockers, calcium-channel blockers, or direct renin inhibitors (previously untreated patients were also eligible in specific circumstances); and had at least one component of the metabolic syndrome in addition to hypertension. Patients with known diabetes were excluded. Patients were randomly assigned (1:1:1) to 24 weeks of daily oral treatment with starting doses of 10 mg amiloride, 25 mg hydrochlorothiazide, or 5 mg amiloride plus 12·5 mg hydrochlorothiazide; all doses were doubled after 12 weeks. Random assignment was done via a central computer system. Both participants and investigators were masked to assignment. Our hierarchical primary endpoints, assessed on a modified intention-to-treat basis at 12 and 24 weeks, were the differences from baseline in blood glucose measured 2 h after a 75 g oral glucose tolerance test (OGTT), compared first between the hydrochlorothiazide and amiloride groups, and then between the hydrochlorothiazide and combination groups. A key secondary endpoint was change in home systolic blood pressure at 12 and 24 weeks. This trial is registered with ClinicalTrials.gov, number NCT00797862, and the MHRA, Eudract number 2009-010068-41, and is now complete.

**Findings:**

Between Nov 18, 2009, and Dec 15, 2014, 145 patients were randomly assigned to amiloride, 146 to hydrochlorothiazide, and 150 to the combination group. 132 participants in the amiloride group, 134 in the hydrochlorothiazide group, and 133 in the combination group were included in the modified intention-to-treat analysis. 2 h glucose concentrations after OGTT, averaged at 12 and 24 weeks, were significantly lower in the amiloride group than in the hydrochlorothiazide group (mean difference −0·55 mmol/L [95% CI −0·96 to −0·14]; p=0·0093) and in the combination group than in the hydrochlorothiazide group (−0·42 mmol/L [–0·84 to −0·004]; p=0·048). The mean reduction in home systolic blood pressure during 24 weeks did not differ significantly between the amiloride and hydrochlorothiazide groups, but the fall in blood pressure in the combination group was significantly greater than that in the hydrochlorothiazide group (p=0·0068). Hyperkalaemia was reported in seven (4·8%) patients in the amiloride group and three (2·3%) patients in the combination group; the highest recorded potassium concentration was 5·8 mmol/L in a patient in the amiloride group. 13 serious adverse events occurred but the frequency did not differ significantly between groups.

**Interpretation:**

The combination of amiloride with hydrochlorothiazide, at doses equipotent on blood pressure, prevents glucose intolerance and improves control of blood pressure compared with montherapy with either drug. These findings, together with previous data about morbidity and mortality for the combination, support first-line use of amiloride plus hydrochlorothiazide in hypertensive patients who need treatment with a diuretic.

**Funding:**

British Heart Foundation and National Institute for Health Research.

## Introduction

The optimum diuretic for hypertension remains uncertain. Disparity has been growing between the drugs and doses proven to reduce risk of stroke, myocardial infarction, and heart failure, and those recommended by guidelines.^1^, ^2^ This move away from recommendation of diuretics in guidelines was driven by an awareness that thiazide and thiazide-like diuretics can increase risk of developing type 2 diabetes.^3^, ^4^, ^5^, ^6^, ^7^ The risk seems linked to potassium depletion, and might be avoided by use of potassium-sparing diuretics,^3^, ^4^, ^8^ which are conventionally thought to be the weakest class of diuretic because most filtered sodium is reabsorbed upstream of their site of action in the nephron. But potassium-sparing diuretics target a common site of sodium retention in hypertension, and might be essential in the prevention of compensatory responses to the more proximally acting thiazide and loop diuretics.^9^ Thus, the hypothesis arose for the present study that an adequate dose of potassium-sparing diuretic would have opposite effects on potassium and glucose to those of a thiazide diuretic, but would have similar or additional effects on blood pressure when the two were compared or combined.

Panel **Research in context**

**Evidence before this study**

We searched MEDLINE and Ovid with the terms “thiazide diuretic”, “potassium”, and “glucose tolerance” under the medical subject headings “diabetes” and “hypertension” for observational studies or clinical trials published in English of diuretic use, diabetes, and glucose tolerance in hypertension. We did our last search on July 23, 2015. A network meta-analysis by Elliott and Meyer of incident diabetes in 22 clinical trials of antihypertensive drugs involving 143 153 participants showed that placebo groups had a lower odds ratio of developing diabetes (0·77, 95% CI 0·63–0·94) when compared with thiazide-assigned groups. In a retrospective meta-analysis by Zillich and colleagues, hypokalaemia and hyperglycaemia were significantly associated in patients given thiazide diuretics. There was an average reduction in serum potassium of 0·23 mmol/L and an increase in glucose of 3·26 mg/dL in studies in which potassium supplements or potassium-sparing drugs were used. In studies in which potassium supplements or potassium-sparing drugs were not used, the average reduction in serum potassium concentrations was 0·37 mmol/L, and the increase in serum glucose was 6·01 mg/dL (p<0·03). A quantitative review by the National Heart, Lung, and Blood Institute in 2008 cast some doubt on the strength of the observational studies that first led to recognition of the thiazide association with diabetes, and investigators noted that the strongest evidence for an adverse effect of thiazides was in prospective outcome comparisons with placebo. The conclusion was that prospective studies should be done to investigate the hypothesis that hypokalaemia is the mediator of thiazide-induced dysglycaemia. Since 2008, diuretic doses and use have fallen because of concerns about metabolic consequences.

**Added value of this study**

In our study we compared a potassium-sparing diuretic, a potassium-losing diuretic, and a combination of the two during 24 weeks in 441 patients who were either previously untreated or taking angiotensin-converting enzyme inhibitors, angiotensin-receptor blockers, or calcium-channel blockers. Our results supported our hypothesis that thiazide-induced glucose intolerance, as shown by 2 h glucose concentrations in oral glucose tolerance tests, would not occur in the absence of potassium depletion. Our results do not completely prove that potassium depletion causes the effect of thiazide on glucose tolerance, but show that it is possible to potentiate the benefit of two classes of diuretic on blood pressure while cancelling out undesired effects of thiazides on glucose and potassium concentrations.

**Implications of all the available evidence**

At an adequate dose (10–20 mg), amiloride is as efficacious as 25–50 mg hydrochlorothiazide, and is not associated with undesirable metabolic consequences. The combination of amiloride and hydrochlorothiazide was already known, from the Medical Research Council's Elderly trial and INSIGHT, to be more efficacious than comparator drugs at preventing some complications of hypertension; however, the 2·5–5 mg dose of amiloride given in these studies was inadequate to prevent hydrochlorothiazide-associated hypokalaemia or diabetes. We propose that the amiloride–hydrochlorothiazide combination tested in this study should be considered in patients taking either an angiotensin-converting enzyme inhibitor, an angiotensin-receptor blocker, or a calcium-channel blocker as a first-line treatment for hypertension.

The prevailing view in the 1990s and 2000s was that, at low doses, thiazides did not cause metabolic consequences but remained maximally efficacious at lowering blood pressure.^10^ Such a view was initially supported by under-powered comparisons of doses, in which no difference in effects on blood pressure were reported.^11^, ^12^ However, an apparent dose–response correlation for blood pressure was rediscovered during treatment titration for an outcome comparison of diuretics with calcium-channel blockers, and this relation was confirmed by a formal, crossover comparison of doses in the Spironolactone, Amiloride, Losartan, Thiazide (SALT) study.^13^, ^14^ The results of SALT also showed the potential value of potassium-sparing diuretics as an alternative to high-dose thiazides. However, in practice, concerns about hyperkalaemia limit the use of potassium-sparing diuretics, especially in an era when most patients are receiving a renin–angiotensin system (RAS) blocker. Additionally, without proof that potassium-sparing diuretics are not associated with glucose intolerance, or indeed that they prevent major complications of hypertension, drugs such as spironolactone have not been included as options before stage 4 hypertension in most national guidelines, still less a replacement for thiazides as first-line treatment.^11^, ^12^, ^15^, ^16^

In combination with a thiazide diuretic, however, amiloride has been used for many years, and in two studies^13^, ^17^ of morbidity and mortality, primary or secondary outcomes were significantly better with an amiloride–hydrochlorothiazide combination than with a calcium-channel blocker or a β blocker. But the low dose of amiloride in the widely available fixed-dose combination used in these studies is insufficient to prevent hypokalaemia. Furthermore, the overall dose of amiloride–hydrochlorothiazide needed to achieve the same reduction in blood pressure produced by the use of a calcium-channel blocker resulted in a 25% excess of new-onset diabetes in a previous trial.^13^

In the Study of Trandolapril/verapamil SR [sustained release] and Insulin Resistance (STAR),^18^ 200 obese patients were randomly assigned to receive either an angiotensin-converting enzyme (ACE) inhibitor plus a calcium-channel blocker or an angiotensin-receptor blocker (ARB) plus a low-dose thiazide diuretic. After 12 weeks, the ARB–diuretic group had significantly higher 2 h glucose concentrations on an oral glucose tolerance test (OGTT). Furthermore, pilot studies^19^, ^20^ showed that high doses of amiloride might be safely used in patients taking a RAS blocker, and have neutral or beneficial effects on glucose tolerance. These studies informed the duration and size of our three-way, randomised, parallel-group study, the British Hypertension Society's Prevention and Treatment of Hypertension with Algorithm-based Therapy (PATHWAY) 3 study, in which we compared the effects of hydrochlorothiazide with those of amiloride, either alone or in combination, on glucose tolerance and blood pressure.

## Methods

### Study design and participants

PATHWAY-3 was a 24 week, parallel-group, randomised, double-blind, phase 4 trial done by the British Hypertension Society Research Network of Investigators at 11 secondary care and two primary care centres in the UK. The study design and rationale have been published previously (appendix).^21^

Eligible patients were aged 18–80 years and had clinic systolic blood pressure of 140 mmHg or higher, home systolic blood pressure of 130 mm Hg or higher, an indication for diuretic treatment, such as high systolic pressure despite treatment with an RAS blocker, and at least one component of the metabolic syndrome in addition to hypertension. Any permutation of ACE inhibitors, ARBs, β blockers, calcium-channel blockers, and direct renin inhibitors was permitted as background treatment, which could be changed at the patient's screening visit for inclusion, but not thereafter. Patients were permitted to take drugs for other disorders, with some specific exceptions (appendix). Diuretic treatment at screening was permitted if it could be discontinued during the 1 month run-in period and replaced with the randomly allocated diuretic. Patients who had not previously been treated for hypertension were eligible for inclusion provided that they were older than 55 years or black, or had a plasma renin concentration of less than 12 mU/L, or any combination thereof. Key exclusion criteria were diabetes diagnosed before enrolment, estimated glomerular filtration rate of less than 45 mL/min per 1·73m^2^, and plasma potassium concentrations outside normal ranges (appendix). A full list of inclusion and exclusion criteria is provided in the appendix. All patients gave informed written consent. The protocol was approved by Cambridge South Ethics Committee.

### Randomisation and masking

After a month's placebo run-in, during which patients were masked to treatment, enrolled patients were randomly assigned (1:1:1) to 10 mg amiloride hydrochloride force-titrated to 20 mg, 25 mg hydrochlorothiazide force-titrated to 50 mg, or a combination of 5 mg amiloride plus 12·5 mg hydrochlorothiazide force-titrated to 10/25 mg orally daily. Doses were doubled in a single step after 12 weeks of treatment. Treatments were allocated according to randomly permuted blocks of size six with each treatment group appearing twice in each block. Randomisation was achieved via a computer-generated list of pseudo-random numbers in the Robertson Centre for Biostatistics (University of Glasgow, UK), which was the data management centre for the study. Complete blocks of treatment packs were allocated to study sites, thus ensuring balanced allocation of treatments within sites and over time. Treatment allocation was done by study nurses, who accessed a web-based randomisation system on a server in the Robertson Centre for Biostatistics.

All trial drugs were packed in identical containers by Alan Wong and colleagues at the Royal Free Hospital Pharmacy, London, UK, and labelled only by subject number and study phase. Investigators, laboratory staff, and patients were masked to the identity of drugs (although the individual drugs, amiloride and hydrochlorothiazide, had a different appearance), and to their sequence allocation. All other antihypertensive drugs already being taken at the time of randomisation were continued unchanged and open-label.

### Procedures

After randomisation, patients took the initial doses of their assigned drug for 12 weeks (phase 1). The doses were then doubled, and treatment continued for a further 12 weeks (phase 2). An OGTT was done at baseline, 12 weeks, and 24 weeks. Blood was taken at 0, 30, 60, and 120 min after administration of a 75 g glucose drink; glucose was tested at all timepoints and insulin at 0 and 30 min.

Other measurements were done at the same timepoints as the OGTTs. Seated home and clinic blood pressure were measured for each patient with their allocated, approved, automated blood pressure monitor (WatchBP Home, Microlife; Clearwater, FL, USA) for the duration of the trial. Patients took home blood pressure readings in the morning and the evening in triplicate on 4 consecutive days before each OGTT. Participants were instructed by specialist nurses in the use of the monitor, and training was reinforced at each clinic visit at which seated clinic blood pressure was measured—the mean of the last two measurements (three were taken). Before each OGTT, patients were also weighed, and had blood drawn for measurement of renin concentrations (analysed by the Diasorin Liaison automated chemiluminescent immunoassay for direct renin mass; Gerenzano, Italy^22^) and other biochemical parameters.

### Outcomes

The primary outcome was the change from baseline in plasma glucose concentrations 2 h after oral administration of a 75 g glucose drink at 12 and 24 weeks.

The main secondary outcome was the change in home systolic blood pressure from baseline at at 12 and 24 weeks. Other secondary endpoints were changes in clinic systolic blood pressure, weight, electrolytes and calcium, uric acid, renin (as measure of natriuresis), HbA_1c_, insulin (at 0 and 30 min during OGTT), area under the curve for glucose during OGTT, and lipid profile. Development of diabetes was defined by fasting glucose concentrations ≥7 mmol/L or 2 h glucose concentrations ≥11·1 mmol/L or HbA_1c_ ≥6·5% (47·5 mmol/mol).

Adverse events were recorded in free text at each visit, and coded by the data management centre on the basis of the medical dictionary for regulatory activities. Serious adverse events were documented and reported to the chief investigator and regulatory authorities, in accordance with local and national requirements.

### Statistical analysis

Based on at least 80% power to detect a mean difference in 2 h glucose between two treatment groups of 1 mmol/L (SD 2·2) with two-sample *t* tests with a 1% significance level, 414 patients were needed. 1 mmol/L was the observed difference in 2 h glucose concentrations in the largest previous trial of glucose intolerance caused by hydrochlorothiazide.^18^

To test both the mechanistic hypothesis—ie, that a potassium-sparing diuretic (amiloride) is better than a potassium-losing diuretic (hydrochlorothiazide) in terms of effect on glucose tolerance—and that their combination is a practical alternative to hydrochlorothiazide monotherapy, the study had two hierarchical primary endpoints: we first compared 2 h glucose in the hydrochlorothiazide group with that of the amiloride group. If the difference between the groups was significant, we then compared 2 h glucose in the hydrochlorothiazide group with the combination amiloride–hydrochlorothiazide group. Hierarchical analysis prevents loss of power, because the prespecified first test needs to be positive before the next can be examined.

We tested for differences between groups for primary and secondary endpoints by using mixed-effect models to analyse continuous variables, with unstructured covariances for repeated measures within a patient, and adjustments for prespecified baseline covariates (sex, age, height, weight, smoking history, and the baseline value of the outcome being analysed). We estimated least-squares means for each treatment from these models, which are averaged from measurements at 12 and 24 weeks unless stated otherwise. We used similar models to assess baseline measurements that predicted response in 2 h glucose and home systolic blood pressure. We used logistic models to compare the proportion of patients who achieved target systolic blood pressure (defined as ≤140 mm Hg) at 24 weeks between the treatment groups and to compare the proportion of patients who developed diabetes by the end of the study between the three treatment groups. We used Fisher's exact test for comparisons of adverse events between groups.

We used SAS (version 9.3) for our data analyses for the modified intention-to-treat population, which included all randomly assigned participants except for those with no primary outcome data from any follow-up visits. We included other participants for whom data were missing, and assumed that data were missing at random (ie, its absence was unrelated to the unobserved value). We did sensitivity analyses in the per-protocol population, which included participants who completed all follow-up visits with no major protocol deviation (adjudicated before breaking the study masking). The safety population, in whom the rate of adverse events and withdrawals was determined, was all randomised patients. There was no data monitoring board. This trial is registered with Clinicaltrials.gov, number NCT00797862 and the MHRA, Eudract number 2009-010068-41.

### Role of the funding source

The funders of the study had no role in study design (other than through the peer-review process), the collection, analysis, or interpretation of the data, or the writing of the report. The corresponding author had full access to all the data and the final responsibility to submit for publication.

## Results

**Figure 1:**
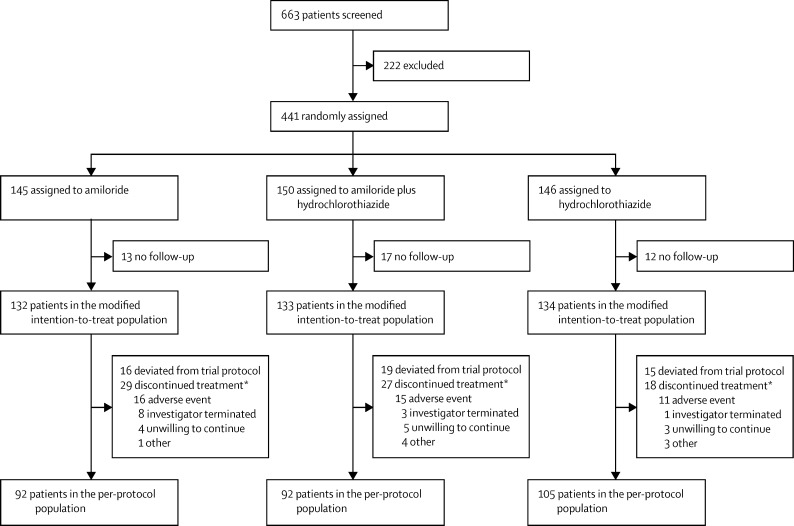
Trial profile Patients who underwent at least one follow-up observation for the primary endpoint after randomisation were included in the modified intention-to-treat analyses. The appendix lists reasons for non-randomisation, dropout between randomisation and modified intention-to-treat populations, and “other” reasons for discontinuation of randomly assigned treatment. Reasons for exclusion from the per-protocol cohort are not mutually exclusive. *Some discontinuations were also protocol deviations, so the differences between modified intention-to-treat and per-protocol populations are less than sum of protocol deviations and discontinuations.

Between Nov 18, 2009, and Dec 15, 2014 we screened 663 patients and randomly assigned 441. 145 patients were assigned to the amiloride group, 146 to the hydrochlorothiazide group, and 150 to the amiloride–hydrochlorothiazide combination group (figure 1). The modified intention-to-treat analysis included 132 patients in the amiloride group, 134 patients in the hydrochlorothiazide group, and 133 patients in the amiloride plus hydrochlorothiazide group (figure 1; appendix).

Table 1 shows baseline characteristics of the modified intention-to-treat population. The commonest indication for diuretic therapy was blood pressure uncontrolled by an ACE inhibitor or ARB, with more than 85% of participants in each group already taking these drugs. The commonest other component of the metabolic syndrome was central obesity, which was present in 99% of subjects.

The mean change from baseline in plasma glucose concentration at the 2 h timepoint during OGTT was −0·55 mmol/L (95% CI −0·96 to −0·14; p=0·0093) in the amiloride group versus the hydrochlorothiazide group when 12 week and 24 week measurements were averaged (table 2). Because this difference was significant, we then examined the other hierarchical primary endpoint: mean change from baseline in 2 h plasma glucose concentration in the combination group was significantly lower than that in the thiazide group (−0·42 [–0·84 to −0·004; p=0·048).

**Figure 2:**
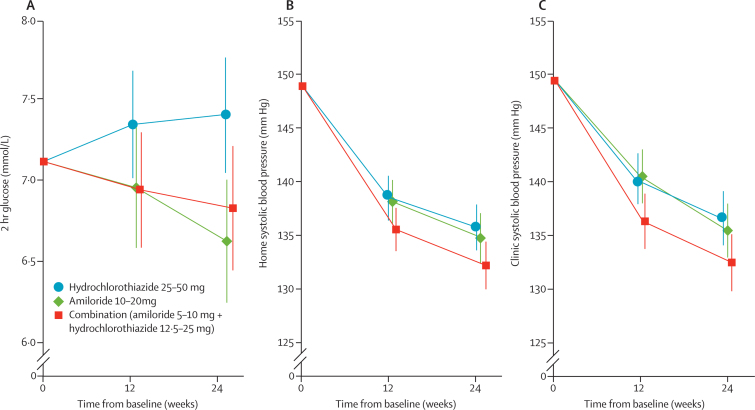
Changes in 2 h blood glucose concentrations (A), home systolic blood pressure (B), and clinic systolic blood pressure (C) Data are adjusted means; error bars show 95% CIs. For (A), p=0·0026 for the comparison between the amiloride and hydrochlorothiazide groups and 0·039 for comparisons between the combination and hydrochlorothiazide groups at 24 weeks, in a model adjusting for baseline covariates. For (B), averaged across 12 weeks and 24 weeks, the fall in home blood pressure was significantly greater in the combination group than in the hydrochlorothiazide group (p=0·0068). For (C), averaged across 12 weeks and 24 weeks, the fall in clinic blood pressure was significantly greater in the combination group than in the hydrochlorothiazide group (p=0·0064).

**Figure 3:**
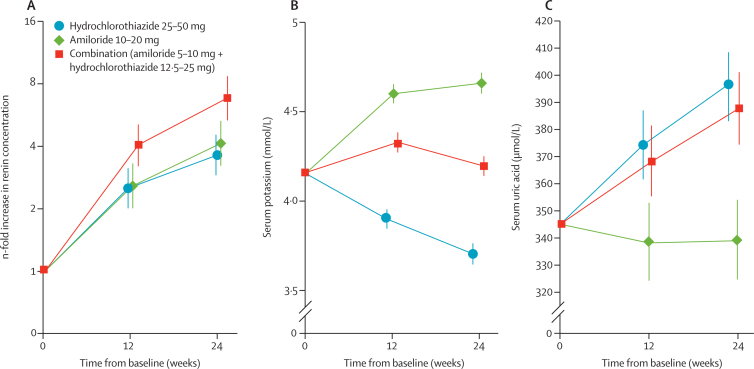
Changes in plasma renin (A), serum potassium (B), and serum uric acid (C) concentrations Data for (A) are log-transformed changes from baseline; data for (B) and (C) are adjusted means. Error bars show 95% CIs. For (A), p=0·0002 for the comparison between the combination and hydrochlorothiazide groups at 24 weeks, from a model adjusting for baseline covariates. For (B), p<0·0001 for the comparison between the amiloride and the hydrochlorothiazide groups and between the combination and hydrochlorothiazide groups at 24 weeks, from a model adjusting for baseline covariates. For (C), p<0·0001 for the comparison between the amiloride and hydrochlorothiazide groups at 24 weeks, from a model adjusting for baseline covariates.

Differences in 2 h glucose concentrations between the hydrochlorothiazide group and the other groups increased with time and dose (figure 2), and similar differences were noted in the per-protocol population (appendix). The mean change in home systolic blood pressure averaged over 24 weeks was −12·9 mm Hg (95% CI −14·7 to —11·2) in the amiloride group, −12·2 mm Hg (−13·9 to −10·5) in the hydrochlorothiazide group, and −15·6 mm Hg (−17·3 to −13·8) in the combination group. The fall in home systolic blood pressure was 3·4 mm Hg (0·9 to 5·8) greater in the combination than in the hydrochlorothiazide group (figure 2B; p=0·0068), averaged over 24 weeks. Mean changes in clinic systolic blood pressure during 24 weeks did not differ significantly between the amiloride group (change from baseline −16·8 mm Hg [–18·8 to −14·8]) and the hydrochlorothiazide group (−16·5 mm Hg [–18·4 to −14·5]). In the combination group, systolic blood pressure fell by −20·4 mm Hg (−22·4 to −18·4), which was a significantly greater reduction compared with the hydrochlorothiazide group (between group difference −3·9 mm Hg [–6·7 to −1·1], p=0·0064). Blood pressures at each visit are shown in table 3. Blood pressure was more likely to be controlled by the combination than by hydrochlorothiazide monotherapy (odds ratio 1·77 [1·05 to 2·96], p=0·031; appendix). The mean rise in renin concentrations was 1·69 (1·36 to 2·01, p<0·0001) times higher at 12 and 24 weeks (average across the two timepoints) in the combination group than in the hydrochlorothiazide group (figure 3A). Significant differences were also noted in other markers of natriuresis, such as urea and creatinine concentrations, but not bodyweight (table 3).

Plasma potassium was unchanged at 24 weeks in the combination group, whereas a significant dose-dependent rise in concentration was noted in the amiloride group (by 0·63 mmol/L [0·56 to 0·70]; p<0·0001 at 24 weeks) and a significant fall was recorded in the hydrochlorothiazide group (by −0·27 mmol/L [–0·34 to −0·20]; p<0·0001 at 24 weeks; figure 3B). Plasma concentrations were significantly higher at 24 weeks in both the amiloride and combination groups than in the hydrochlorothiazide group (p<0·0001 for both when adjusted for baseline covariates; figure 3B).

Uric acid concentrations rose in the hydrochlorothiazide group but were unchanged in the amiloride group (p<0·0001 for difference between groups; table 3, figure 3C); uric acid concentrations did not differ significantly between the hydrochlorothiazide and combination groups. Area under the curve during OGTT was significantly smaller in the amiloride group than in the hydrochlorothiazide group, but did not differ significantly between the combination and hydrochlorothiazide groups (table 3). We noted no significant differences between groups in fasting glucose, insulin (at 0 and 30 min), HbA_1c_, or homoeostatic model assessment indices of insulin resistance and secretion (table 3), except for a difference between the hydrochlorothiazide and combination groups for insulin at 30 min. However, we noted a mean numerical increase across all groups in HbA_1c_ at both 12 weeks (0·086% [0·033–0·139], p=0·0006) and 24 weeks (0·126% [0·082–0·170], p<0·0001). 11 patients in the amiloride (11·8% adjusted for baseline covariates [95% CI 6·3–21·1]), nine (8·0% [3·7–16·2]) in the combination, and 13 (12·6% [6·5–23·0]) in the hydrochlorothiazide group developed diabetes during the study. Odds ratios for developing diabetes compared with the hydrochlorothiazide group were 0·65 (0·25–1·69) for the combination group and 1·07 (0·43–2·64) for the amiloride group.

Predictors of the glucose and blood pressure responses to study drugs are shown in the appendix. For change from baseline in 2 h glucose concentrations the main predictors were baseline fasting or 2 h glucose concentrations. For blood pressure, the main predictors were baseline blood pressure and plasma renin concentration. Blood glucose at the end of the study was weakly and inversely correlated with serum potassium concentrations (N=314, *r*^2^=0·02, p=0·010; no differences between treatments [p=0·60]; appendix).

All drugs were well tolerated. 13 serious adverse events were reported. All adverse events are listed in the appendix. Only dizziness and muscle spasms were recorded in nine or more participants in each group; frequency of these events did not differ significantly between groups (table 4). Hyperkalaemia was reported in ten patients receiving amiloride alone or with hydrochlorothiazide (table 4). However the highest recorded potassium concentration was 5·8 mmol/L (in the amiloride group), and most values were between 5·1 mmol/L and 5·3 mmol/L (data not shown) in patients at a site where 5·0 was the upper limit of the normal laboratory range (appendix).

## Discussion

Diuretics have been used as the control drug in many large hypertension studies, but have rarely in the past 10 years been the main target of interest or studied in maximally efficacious doses.^23^, ^24^ Our study provides answers to several questions about diuretics that had not been previously investigated or resolved. We showed that, after 24 weeks of treatment, a potassium-sparing diuretic reduces blood pressure as efficaciously as high-dose thiazide without inducing adverse effects on blood glucose concentrations. Furthermore, a combination of half the conventional doses of amiloride and hydrochlorothiazide was not associated with increased 2 h glucose concentrations compared with hydrochlorothiazide treatment alone but produced significantly larger reductions in blood pressure than full doses of either diuretic given alone. Amiloride monotherapy did not cause clinically significant hyperkalaemia, and the amiloride–hydrochlorothiazide combination did not significantly affect potassium concentrations.

Hitherto, the mechanism and prospects for prevention of thiazide-induced glucose intolerance were uncertain; the role of potassium in this problem was also unclear. A National Heart, Lung, and Blood Institute working party in 2008 identified potassium as “perhaps the most attractive variable” in developing a hypothesis for the mechanism of the thiazide response, and called for studies of potassium-sparing diuretics, among others.^6^ Amiloride has been licensed for hypertension for almost as long as hydrochlorothiazide, but has rarely been used or studied in doses that lower blood pressure as effectively as high-dose thiazides or other diuretic classes.^25^ That matched doses of thiazides and potassium-sparing diuretics, with similar efficacy on blood pressure, could neutralise the undesirable effects of each class while synergising to enhance reduction of blood pressure was an attractive hypothesis, but there were many unknowns, such as whether amiloride—in the context of blockade of the RAS in most patients—could be safely used at a dose large enough to match the blood pressure reduction of hydrochlorothiazide without causing hazardous electrolyte abnormalities.

Glucose concentration at 2 h in the OGTT is the best single measure for prediction of the long-term development of diabetes,^26^, ^27^, ^28^ and is also a strong predictor of cardiovascular morbidity.^29^ Sequential OGTTs offered the possibility of testing the hypothesis that prevention of potassium depletion would protect against thiazide-induced glucose intolerance. Although some of the difference in glucose profiles between the groups was due to a progressive increase in glucose intolerance in the hydrochlorothiazide group, glucose concentrations fell significantly in the amiloride group, at least when 12 week and 24 week data were compared with baseline. The importance of even minor degrees of glucose intolerance has been long evident from the Whitehall study,^30^ which showed that, during a period of 7·5 years, mortality from coronary heart disease doubled in participants with a 2 h glucose concentration greater than 5·3 mmol/L compared with patients with 2 h concentations of less than 5·3 mmol/L.

That amiloride and hydrochlorothiazide have opposite effects on glucose tolerance is consistent with potassium depletion being the cause of the rise in blood glucose concentration in patients taking thiazide diuretics. The poor correlation in our study between plasma glucose and potassium might be related to how poorly plasma electrolytes reflect overall electrolyte balance—plasma potassium concentrations are often normal in primary aldosteronism, for example.^31^ However, we included an intermediate, combination group in the study, both to confirm the role of potassium and to investigate a treatment that could be implemented in practice. The combination group necessitated selection of doses of each drug that, unlike the available fixed-dose combinations (in which the dose of amiloride is only a tenth that of hydrochlorothiazide), were predicted to neutralise changes in potassium. If the effects on blood glucose concentrations in the combination group were, as we noted with potassium concentrations, halfway between those in the two monotherapy groups, an even larger study than PATHWAY-3 would have been needed. But our additional predictions were that blood pressure would trump other effects on blood glucose, and that combining half doses of two diuretics with different targets in the kidney would have synergistic effects on sodium excretion, hence leading to reduction in blood pressure.

Our prediction of natriuretic synergism seems to be confirmed by the significantly greater reduction in home and clinic systolic blood pressure in the combination group than in the hydrochlorothiazide group. The blood pressure reduction in the combination group was associated with a near-doubling of the reactive rise in renin concentrations compared with those in the other two groups. This rise in renin concentrations is not clinically important in patients taking RAS blockers, but is a sensitive measure of natriuresis.^32^ Results of cross-sectional studies^33^, ^34^ have suggested the importance of blood pressure control in the prevention of glucose intolerance, which might be underestimated in comparisons of different antihypertensive drug classes. In studies of single antihypertensive drugs versus placebo, the reduced incidence of diabetes might be ascribed to the specific class effect rather than reductions in blood pressure reduction.^35^ The results of our pilot crossover studies showed that drugs that impair glucose tolerance when used alone (ie, β blockers and thiazide diuretics) have a neutral effect on glucose tolerance when combined with each other, and that the more favourable effects of the combination (compared with monotherapy) on glucose tolerance is associated with superior blood pressure reduction.^19^ Therefore the more efficacious blood pressure reduction in the amiloride–hydrochlorothiazide group compared with either drug alone is probably what underpins the success of the combination in avoiding induction of glucose intolerance.

The main limitation of our study is the short duration. 24 weeks was long enough to test the hypotheses that drugs with opposite effects on potassium concentrations would have opposite effects on 2 h glucose concentrations, and that diuretics with different sites of action would have synergistic effects on sodium loss, leading to blood pressure reduction, leading to glucose tolerance. However, not all patients who had a rise in 2 h glucose concentration in our study will progress to diabetes, and our results cannot show that longer-term usage of potassium-sparing diuretics will prevent an increase in incidence of diabetes as that which occurs with thiazide diuretics. Furthermore, 24 weeks' exposure is not sufficient to provide complete assurance of long-term safety when amiloride is added to an RAS blocker—intermittent monitoring of electrolytes and estimated glomerular filtration rates will be necessary in practice.

The short duration of the study also limits our ability to explain why potassium-sparing and potassium-losing diuretics have opposing effects on blood glucose. On several measures—insulin concentrations, HbA_1c_, and the calculated homoeostatic model assessments of insulin secretion and resistance—we found no differences between groups. Good control of blood pressure and the high prevalence of ACE inhibitors or ARBs in our study population probably mitigated the rise in 2 h blood glucose in the hydrochlorothiazide group, despite the high proportion of patients with central adiposity.^33^, ^34^, ^35^ But results in the amiloride group—in which there were seemingly contrary trends for HbA_1c_ and 2 h glucose concentrations—show previously noted limitations of using HbA_1c_ as a surrogate of glucose intolerance.^36^ Thus, the adverse effect of spironolactone on glucose tolerance, which was imputed from a similar small rise in HbA_1c_, could possibly be an artifact.^37^ The mechanism to explain why HbA_1c_ does not accurately reflect glucose intolerance in this setting can only be speculated, but perhaps redistribution and reduction of blood supply by diuretics affects red cell disposal and hence the turnover of HbA_1c_. A final limitation is that we enrolled patients with an increased likelihood of developing glucose intolerance. The beneficial effects of amiloride might be less certain or applicable in thinner patients.

Diuretics are the oldest among commonly used antihypertensive drugs, and their target (sodium retention) is one of the few universally agreed contributors to the pathogenesis of hypertension.^1^ Yet they have slipped in priority in some guidelines, partly because of trials in which suboptimum doses of diuretics were compared with optimum doses of other classes, and partly because of thiazide-induced diabetes. Even if thiazide-induced diabetes does not carry the same cardiovascular risks as spontaneous diabetes, diuretics cease to be cost effective when extra clinic visits and treatments for diabetes are factored into analyses.^38^ On the basis of our results from PATHWAY-3, we recommend that the combination of amiloride and hydrochlorothiazide, in doses equipotent for blood pressure reduction, becomes the first-choice diuretic in patients in whom adequate diuretic has not yet been prescribed. Our results suggest that this drug combination will confer the proven long-term benefits of hydrochlorothiazide without the possible downside of glucose intolerance. Low doses of amiloride with hydrochlorothiazide in the INSIGHT trial^13^ were as efficacious as nifedipine in the prevention of stroke and myocardial infarction, and significantly more efficacious in the prevention of heart failure. In the Medical Research Council's Elderly trial,^17^ the combination was significantly superior to atenolol in all cardiovascular endpoints.^17^ On the basis of our findings, the combination of hydrochlorothiazide with four times as much amiloride as was used in these studies can be predicted to increase its antihypertensive efficacy and counter the risks and costs of hypokalaemia and glucose intolerance. The efficacy of potassium-sparing diuretics revealed by PATHWAY-3, and the parallel PATHWAY-2 study of spironolactone in patients with resistant hypertension,^39^ warrants investigation of their long-term benefits in hypertension.

## Figures and Tables

**Table 1:** Baseline characteristics in the modified intention-to-treat population

		**Amiloride (n=132)**	**Amiloride plus hydrochlorothiazide (n=133)**	**Hydrochlorothiazide (n=134)**
Age (years)		62·1 (10·4)	61·5 (10·2)	62·8 (9·9)
Female sex		52 (39·4%)	63 (47·4%)	47 (35·1%)
Weight (kg)		89·3 (16·7)	88·8 (16·7)	88·2 (17·1)
BMI (kg/m^2^)		31·4 (7·6)	31·0 (4·7)	30·6 (5·1)
Number of current smokers		10 (7·6%)	12 (9·0%)	15 (11·2%)
Blood pressure (mm Hg)				
	Clinic systolic	153·8 (11·4)	156·2 (12·4)	154·4 (11·7)
	Clinic diastolic	91·3 (9·7)	91·2 (9·4)	90·0 (10·2)
	Home systolic	149·3 (12·4)	150·6 (11·4)	148·8 (10·9)
	Home diastolic	86·9 (9·8)	86·6 (8·9)	85·1 (9·6)
Previously untreated		7 (5·3%)	12 (9·0%)	11 (8·2%)
Receiving ACE inhibitor or ARB		119 (90·2%)	115 (86·5%)	117(87·3%)
Receiving β blocker		18 (13·6%)	24 (18·0%)	23 (17·2%)
Receiving calcium-channel blocker		56 (42·4%)	57 (42·9%)	56 (41·8%)
Number of drugs (if treated)		1·5 (0·7)	1·5 (0·7)	1·6 (0·7)
Central obesity*		129 (97·7%)	133 (100·0%)	132 (98·5%)
Serum potassium (mmol/L)		4·1 (0·4)	4·2 (0·3)	4·2 (0·4)
2 hour glucose during OGTT (mmol/L)		7·2 (2·3)	7·2 (2·1)	6·9 (2·4)
Impaired glucose tolerance†		44 (33·3%)	45 (33·8%)	42 (31·3%)

Data are mean (SD) or n (%). ACE=angiotensin-converting enzyme. ARB=angiotensin-receptor blocker. OGTT=oral glucose tolerance test.

* Central obesity is defined as a waist circumference of greater than 94 cm in men and greater than 80 cm in women.

† Impaired glucose tolerance is defined as a 2 h glucose concentration of greater than 7·8 mmol/L.

**Table 2:** Changes from baseline in 2 h glucose concentrations during oral glucose tolerance tests in the modified intention-to-treat population

	**Amiloride (n=132)**	**Amiloride plus hydrochlorothiazide (n=133)**	**Hydrochlorothiazide (n=134)**
Mean change from baseline (mmol/L)	−0·35 (−0·69 to −0·01)	−0·22 (−0·56 to 0·11)	0·20 (−0·12 to 0·51)
Difference from hydrochlorothiazide (mmol/L)	−0·55 (−0·96 to −0·14; p=0·0093)	−0·42 (−0·84 to 0·00; p=0·048)	..

Data in parentheses are 95% CIs. Mean change from baseline was calculated on the basis of data at 12 weeks (low-dose treatment) and 24 weeks (high-dose treatment). Least squares estimates were adjusted for prespecified baseline covariates in a mixed-effects model; p values are for comparisons with hydroclorothiazide.

**Table 3:** Changes in blood pressure and biochemical parameters in the modified intention-to-treat population

	**Unadjusted treatment means (95% CI)**			**Adjusted treatment differences (95% CI; p)**	
	Amiloride 10–20 mg	Combination amiloride/hydrochlorothiazide 5 mg/12·5 mg–10 mg/25 mg	Hydrochlorothiazide 25–50 mg	Amiloride *vs* hydrochlorothiazide	Combination *vs* hydrochlorothiazide
**Home systolic blood pressure (mm Hg)**					
Baseline	149·3 (147·2 to 151·5)	150·6 (148·6 to 152·6)	148·8 (146·9 to 150·6)	..	..
12 weeks	138·3 (136·0 to 140·5)	136·1 (133·8 to 138·3)	138·5 (136·6 to 140·5)	−0·5 (−3·2 to 2·1; p=0·70)	−3·2 (−5·8 to −0·5; p=0·020)
24 weeks	134·4 (132·3 to 136·5)	132·3 (130·0 to 134·6)	135·0 (133·0 to 137·0)	−1·0 (−3·7 to 1·7; p=0·49)	−3·5 (−6·2 to −0·8; p=0·011)
**Clinic systolic blood pressure (mm Hg)**					
Baseline	153·8 (151·9 to 155·8)	156·2 (154·0 to 158·3)	154·4 (152·4 to 156·4)	..	..
12 weeks	140·8 (138·5 to 143·1)	136·7 (134·2 to 139·3)	140·3 (137·7 to 142·9)	0·3 (−2·9 to 3·5; p=0·84)	−3·9 (−7·1 to −0·7; p=0·016)
24 weeks	135·4 (132·9 to 137·9)	133·4 (131·0 to 135·8)	135·8 (133·3 to 138·2)	−1·0 (−4·3 to 2·3; p=0·55)	−4·0 (−7·2 to −0·7; p=0·018)
**Bodyweight (kg)**					
Baseline	89·3 (86·4 to 92·1)	88·8 (85·9 to 91·7)	88·2 (85·3 to 91·1)	..	..
12 weeks	90·5 (87·6 to 93·5)	89·1 (86·2 to 92·1)	88·9 (85·9 to 91·8)	0·2 (−0·5 to 0·8; p=0·60)	−0·2 (−0·8 to 0·5; p=0·64)
24 weeks	89·1 (85·9 to 92·2)	89·8 (86·4 to 93·2)	87·2 (84·3 to 90·0)	−0·2 (−0·8 to 0·5; p=0·63)	−0·1 (−0·8 to 0·6; p=0·82)
**Renin (mU/L, log base 10)**					
Baseline	1·20 (1·09 to 1·31)	1·14 (1·03 to 1·24)	1·20 (1·11 to 1·30)	..	..
12 weeks	1·67 (1·53 to 1·80)	1·75 (1·61 to 1·89)	1·62 (1·50 to 1·74)	0·03 (−0·10 to 0·16; p=0·63)	0·21 (0·08 to 0·34; p=0·0018)
24 weeks	1·83 (1·70 to 1·95)	1·95 (1·82 to 2·09)	1·74 (1·62 to 1·86)	0·06 (−0·07 to 0·20; p=0·34)	0·25 0·12 to 0·39; p=0·0002)
**Sodium (mmol/L)**					
Baseline	139·7 (139·3 to 140·2)	139·9 (139·5 to 140·4)	139·7 (139·2 to 140·2)		..
12 weeks	138·2 (137·8 to 138·6)	138·1 (137·7 to 138·5)	138·9 (138·5 to 139·3)	−0·7 (−1·3 to −0·1; p=0·03)	−1·1 (−1·7 to −0·4; p=0·0010)
24 weeks	138·1 (137·7 to 138·5)	138·0 (137·5 to 138·4)	138·6 (138·1 to 139·0)	−0·6 (−1·2 to 0·1; p=0·097)	−0·9 (−1·6 to −0·2; p=0·0075)
**Potassium (mmol/L)**					
Baseline	4·09 (4·01 to 4·16)	4·16 (4·10 to 4·22)	4·21 (4·14 to 4·28)	..	..
12 weeks	4·55 (4·50 to 4·61)	4·31 (4·26 to 4·36)	3·97 (3·92 to 4·03)	0·68 (0·60 to 0·76; p<0·0001)	0·42 (0·34 to 0·50; p<0·0001)
24 weeks	4·61 (4·56 to 4·66)	4·17 (4·12 to 4·23)	3·79 (3·74 to 3·84)	0·90 (0·81 to 0·98; p<0·0001)	0·48 (0·39 to 0·56; p<0·0001)
**Urea (mmol/L)**					
Baseline	5·36 (5·12 to 5·60)	5·15 (4·92 to 5·38)	5·40 (5·15 to 5·65)	..	..
12 weeks	5·90 (5·68 to 6·12)	6·08 (5·85 to 6·32)	6·03 (5·82 to 6·25)	−0·02 (−0·31 to 0·28; p=0·91)	0·34 (0·05 to 0·64; p=0·024)
24 weeks	5·82 (5·59 to 6·06)	6·21 (5·98 to 6·44)	6·13 (5·87 to 6·39)	−0·17 (−0·48 to 0·13; p=0·26)	0·45 (0·14 to 0·76; p=0·0042)
**Creatinine (μmol/L)**					
Baseline	77·4 (74·4 to 80·4)	76·6 (73·3 to 79·9)	76·7 (73·8 to 79·6)	..	..
12 weeks	79·8 (77·5 to 82·2)	80·1 (77·6 to 82·7)	79·3 (76·9 to 81·6)	1·1 (−1·5 to 3·7; p=0·40)	2·3 (−0·4 to 4·9; p=0·089)
24 weeks	79·7 (77·2 to 82·3)	80·3 (77·7 to 83·0)	78·1 (75·6 to 80·6)	2·9 (0·2 to 5·6; p=0·03	3·8 (1·1 to 6·5; p=0·0057)
**Uric acid (μmol/L)**					
Baseline	354 (340 to 369)	349 (332 to 365)	342 (324 to 359)	..	..
12 weeks	355 (340 to 371)	365 (347 to 383)	375 (358 to 392)	−32 (−49 to −15; p=0·0003)	−7 (−23 to 10; p=0·43)
24 weeks	351 (335 to 368)	380 (362 to 397)	392 (374 to 410)	−50 (−67 to −33; p<0·0001)	−3 (−20 to 14; p=0·76)
**Total cholesterol (mmol/L)**					
Baseline	1·40 (1·27 to 1·52)	1·53 (1·37 to 1·69)	1·34 (1·23 to 1·46)	..	..
12 weeks	1·51 (1·34 to 1·68)	1·59 (1·42 to 1·76)	1·44 (1·30 to 1·58)	0·11 (−0·06 to 0·27; p=0·21)	0·10 (−0·07 to 0·26; p=0·25)
24 weeks	1·59 (1·40 to 1·77)	1·49 (1·35 to 1·63)	1·54 (1·39 to 1·69)	0·01 (−0·16 to 0·18; p=0·93)	−0·16 (−0·33 to 0·01; p=0·057)
**Triglycerides (mmol/L)**					
Baseline	1·40 (1·27 to 1·52)	1·53 (1·37 to 1·69)	1·34 (1·23 to 1·46)	..	..
12 weeks	1·51 (1·34 to 1·68)	1·59 (1·42 to 1·76)	1·44 (1·30 to 1·58)	0·11 (−0·06 to 0·27; p=0·21)	0·10 (−0·07 to 0·26; p=0·25)
24 weeks	1·59 (1·40 to 1·77)	1·49 (1·35 to 1·63)	1·54 (1·39 to 1·69)	0·01 (−0·16 to 0·18; p=0·93)	−0·16 (−0·33 to 0·01; p=0·057)
**LDL cholesterol (mmol/L)**					
Baseline	2·88 (2·70 to 3·05)	2·94 (2·78 to 3·10)	2·95 (2·79 to 3·11)	..	..
12 weeks	2·87 (2·71 to 3·03)	2·92 (2·75 to 3·09)	2·87 (2·72 to 3·03)	−0·00 (−0·16 to 0·16; p=0·96)	0·07 (−0·09 to 0·23; p=0·3605)
24 weeks	3·05 (2·86 to 3·24)	2·95 (2·77 to 3·13)	2·96 (2·80 to 3·12)	0·10 (−0·06 to 0·26; p=0·22)	0·04 (−0·12 to 0·21; p=0·5849)
**HDL cholesterol (mmol/L)**					
Baseline	1·27 (1·17 to 1·36)	1·33 (1·23 to 1·43)	1·35 (1·24 to 1·45)		..
12 weeks	1·17 (1·05 to 1·28)	1·34 (1·24 to 1·44)	1·35 (1·24 to 1·46)	−0·18 (−0·34 to −0·03; p=0·018)	−0·00 (−0·15 to 0·15; p=1·00)
24 weeks	1·20 (1·08 to 1·32)	1·32 (1·20 to 1·44)	1·35 (1·25 to 1·45)	−0·13 (−0·28 to 0·02; p=0·09)	−0·02 (−0·17 to 0·14; p=0·85)
**Fasting glucose (mmol/L)**					
Baseline	5·20 (5·07 to 5·32)	5·13 (5·04 to 5·23)	5·30 (5·13 to 5·46)	..	..
12 weeks	5·30 (5·13 to 5·47)	5·26 (5·15 to 5·38)	5·38 (5·25 to 5·52)	−0·00 (−0·16 to 0·16; p=0·98)	−0·01 (−0·17 to 0·14; p=0·86)
24 weeks	5·23 (5·08 to 5·37)	5·27 (5·15 to 5·39)	5·39 (5·24 to 5·54)	−0·08 (−0·23 to 0·08; p=0·36)	−0·04 (−0·20 to 0·12; p=0·61)
**2 h glucose (mmol/L)**					
Baseline	7·35 (6·87 to 7·82)	7·21 (6·84 to 7·59)	7·03 (6·62 to 7·44)	..	..
12 weeks	6·97 (6·59 to 7·35)	7·03 (6·66 to 7·41)	7·33 (6·98 to 7·69)	−0·36 (−0·84 to 0·11; p=0·13)	−0·30 (−0·78 to 0·18; p=0·21)
24 weeks	6·73 (6·35 to 7·10)	6·92 (6·55 to 7·30)	7·46 (7·11 to 7·81)	−0·74 (−1·21 to −0·26; p=0·0023)	−0·54 (−1·01 to −0·06; p=0·026)
**Fasting insulin (pmol/L)**					
Baseline	80·1 (65·1 to 95·0)	86·2 (60·8 to 111·5)	95·6 (63·7 to 127·5)	..	..
12 weeks	85·1 (74·4 to 95·7)	121·1 (81·4 to 160·7)	101·9 (81·1 to 122·6)	−17·0 (−46·1 to 12·0; p=0·25)	−0·9 (−29·6 to 27·7; p=0·95)
24 weeks	88·7 (76·3 to 101·1)	107·7 (78·8 to 136·6)	102·1 (75·6 to 128·6)	−17·7 (−47·5 to 12·1; p=0·24)	4·9 (−24·6 to 34·5; p=0·74)
**30 min insulin (pmol/L)**					
Baseline	487 (368 to 605)	427 (379 to 474)	445 (378 to 512)	..	..
12 weeks	547 (448 to 645)	616 (484 to 748)	499 (431 to 568)	14 (−72 to 99; p=0·75)	68 (−14 to 150; p=0·10)
24 weeks	584 (488 to 680)	531 (437 to 626)	465 (397 to 533)	51 (−34 to 136; p=0·24)	88 (2 to 174; p=0·04)
**Homoeostatic model assessment—insulin resistance**					
Baseline	2·70 (2·18 to 3·23)	2·93 (2·00 to 3·86)	3·29 (2·20 to 4·38)	..	..
12 weeks	2·90 (2·49 to 3·31)	4·34 (2·80 to 5·87)	3·57 (2·85 to 4·29)	−0·65 (−1·84 to 0·55; p=0·29)	0·02 (−1·14 to 1·19; p=0·97)
24 weeks	3·01 (2·57 to 3·44)	3·88 (2·54 to 5·22)	3·57 (2·60 to 4·53)	−0·84 (−2·05 to 0·37; p=0·17)	0·17 (−1·03 to 1·37; p=0·78)
**Homoeostatic model assessment—β cells**					
Baseline	122·1 (98·6 to 145·7)	134·2 (103·1 to 165·2)	138·3 (93·1 to 183·4)	..	..
12 weeks	55·6 (43·8 to 67·4)	77·9 (54·0 to 101·8)	62·8 (47·7 to 77·9)	−9·8 (−33·8 to 14·3; p=0·42)	13·8 (−10·2 to 37·8; p=0·26)
24 weeks	63·0 (47·1 to 79·0)	71·0 (54·4 to 87·5)	71·4 (51·0 to 91·8)	−11·6 (−36·8 to 13·5; p=0·36)	−2·9 (−28·0 to 22·3; p=0·82)
**HbA**_1c_**(%)**					
Baseline	5·73 (5·63 to 5·83)	5·63 (5·55 to 5·70)	5·65 (5·57 to 5·74)	..	..
12 weeks	5·81 (5·70 to 5·91)	5·70 (5·62 to 5·78)	5·68 (5·54 to 5·82)	0·05 (−0·07 to 0·18; p=0·39)	0·05 (−0·07 to 0·17; p=0·38)
24 weeks	5·76 (5·64 to 5·88)	5·70 (5·60 to 5·81)	5·75 (5·65 to 5·85)	−0·04 (−0·16 to 0·09; p=0·56)	0·00 (−0·12 to 0·13; p=0·95)
**Calcium (mmol/L)**					
Baseline	2·34 (2·32 to 2·35)	2·33 (2·32 to 2·35)	2·32 (2·30 to 2·33)	..	..
12 weeks	2·30 (2·28 to 2·32)	2·31 (2·29 to 2·33)	2·29 (2·27 to 2·31)	−0·01 (−0·04 to 0·02; p=0·62)	0·02 (−0·01 to 0·05; p=0·22)
24 weeks	2·31 (2·28 to 2·34)	2·32 (2·29 to 2·34)	2·31 (2·28 to 2·34)	−0·01 (−0·04 to 0·02; p=0·46)	0·01 (−0·02 to 0·04; p=0·46)
**Change in area under curve during oral glucose tolerance test**					
Baseline	987 (947 to 1027)	962 (931 to 992)	975 (941 to 1010)	..	..
12 weeks	951 (908 to 994)	965 (925 to 1005)	1005 (965 to 1046)	−56 (−98 to −14; p=0·0087)	−29 (−70 to 12; p=0·17)
24 weeks	930 (891 to 968)	975 (932 to 1018)	990 (947 to 1032)	−60 (−103 to −18; p=0·0052)	−20 (−63 to 23; p=0·37)

Comparisons are adjusted for baseline values. The slight differences for 2 h glucose at week 24 between this table and table 2 arise because missing values were imputed in the calculations used for this table whereas they were omitted in table 2. To convert HbA_1c_ to mmol/mol, multiply the percentages by 10·93 and subtract 23·5. To convert renin to pmol/L, multiply concentration in mU/L by 1·56.

**Table 4:** Adverse events and withdrawals in the modified intention-to-treat population

	**Amiloride (n=145)**	**Amiloride plus hydrochlorothiazide (n=150)**	**Hydrochlorothiazide (n=146)**	**Amiloride***vs***hydrochlorothizaide**	**Combination***vs***hydrochlorothiazide**
Withdrawals	17 (11·7%)	16 (10·7%)	10 (6·8%)	0·16	0·31
Serious adverse events	7 (4·8%)	4 (2·7%)	2 (1·4%)	0·10	0·68
Any adverse event	97 (66·9%)	92 (61·3%)	95 (65·1%)	0·80	0·55
Dizziness	9 (6·2%)	15 (10·0%)	16 (11·0%)	0·21	0·85
Muscle spasms	12 (8·3%)	14 (9·3%)	10 (6·8%)	0·66	0·52
Hyperkalaemia	7 (4·8%)	3 (2·0%)	0	0·0071	0·25

Data are n (%) unless otherwise specified. Fisher's exact test was used to calculate p values.
