# Supplementary material for: Effect of amiloride, or amiloride plus hydrochlorothiazide, versus hydrochlorothiazide on glucose tolerance and blood pressure (PATHWAY-3): a parallel-group, double-blind randomised phase 4 trial
Source: Lancet Diabetes Endocrinol. 2016 Feb;4(2):136–47. doi: 10.1016/S2213-8587(15)00377-0 (PMC4728199; doi:10.1016/S2213-8587(15)00377-0)
Supplement: Supplementary appendix [file mmc1.pdf]

THE LANCET Diabetes & Endocrinology

Supplementary appendix

This appendix formed part of the original submission and has been peer reviewed. We post it as supplied by the authors.

Supplement to: Brown MJ, Williams B, Morant SV, et al, for the British Hypertension Society's Prevention and Treatment of Hypertension with Algorithm-based Therapy (PATHWAY) Studies Group. Effect of amiloride, or amiloride plus hydrochlorothiazide, versus hydrochlorothiazide on glucose tolerance and blood pressure (PATHWAY-3): a parallel-group, double-blind randomised phase 4 trial. *Lancet Diabetes Endocrinol* 2015; published online Oct 19. http://dx.doi.org/10.1016/S2213-8587(15)00377-0.

Online Appendix

Contents	Title	Page
PATHWAY Studies Group		2
Supplementary Table 1	Eligibility criteria	3
Supplementary Table 2	Exclusion criteria	4
Supplementary Table 3	Footnotes to CONSORT diagram<br>(a) Reasons for non-randomisation<br>(b) Reasons for non-inclusion in modified ITT population<br>(c) 'Other' reasons for discontinuation of randomised treatment	5
Supplementary Table 4	Changes in 2-hour glucose	7
Supplementary Table 5	BP Control Rates	8
Supplementary Table 6	Predictors of changes in blood glucose and blood pressure	9
Supplementary Table 7	All serious adverse events	10
Supplementary Table 8	Selected adverse events	11
Supplementary Figure 1	Study Schematic	12
Supplementary Figure 2	Oral glucose tolerance tests	13
Supplementary Figure 3	Glucose vs Potassium	14
Supplementary Figure 4	Stacked histograms for electrolytes and eGFR	15

***PATHWAY Studies Group**

1. Anne Schumann, Jo Helmy, Carmela Maniero, Timothy J Burton, Ursula Quinn, Lorraine Hobbs, Sarah Woods, Carole Bartlett, Jo Palmer (Addenbrooke's Cambridge, UK)
2. David Collier, Nirmala Markandu, Manish Saxena, Anne Zak, Enamuna Enobakhare (William Harvey Institute, QMUL, London, UK)
3. Judith Mackay, Simon A McG Thom, Candida Coughlan, (International Centre for Circulatory Health UCL London, UK)
4. Alison R McGinnis, Evelyn Findlay, Caroline Patterson (Ninewells Hospital & Medical School, Dundee, UK)
5. Adrian G Stanley, Christobelle White, Peter Lacey, Caroline J. Gardiner-Hill. (Glenfield Hospital, Leicester, UK)
6. Sandosh Padmanabhan, Gordon T McInnes, Craig Harrow, Linsay McCallum (Glasgow Cardiovascular Research centre, Glasgow, UK)
7. Vanessa Melville, Iain M MacIntyre, Emma Turtle, Roger Brown, Simon Maxwell (University of Edinburgh Western General Hospital, Edinburgh, UK)
8. Handrean Soran, See Kwok, Karthirani Balakrishnan. (Old Saint Mary's Hospital, Manchester, UK)
9. Richard Hobbs, Rachel Iles, Clare Taylor (Primary Care Clinical Sciences University Birmingham, Birmingham, UK)
10. Khin Swe Myint (Clinical Research and trials Unit, University of East Anglia, Norwich, UK)
11. John Cannon, Sue Hood (Ixworth GP Practice, Ixworth, UK)
12. Andrew Webb, Krzysztof Rutkowski (Cardiovascular Medicine & Diabetes, King's College London, and Clinical Research Facility, St Thomas's Hospital, London, UK)
13. Una Martin (University Hospital Birmingham, Birmingham, UK)
14. Sharon Kean, Robbie Wilson, Richard Papworth (Robertson Centre for Biostatistics and Glasgow CTU, Glasgow, UK)

Supplementary Table 1
Eligibility criteria

1. Age 18-80
2. Diagnosis of hypertension according to BHS criteria
3. Systolic BP on permitted background treatment ≥ 140 mmHg and home BP ≥ 130mmHg.
4. Indication for diuretic treatment as a treatment option for the patient's uncontrolled hypertension :<br><br>(a) Untreated + (age>55 AND/OR Black AND/OR renin<12mU/L)<br><br>OR (b) receiving one or any permutation of the following:<br><br>ACEi, ARB, β-blocker, CCB, direct renin inhibitor
5. At least one other component (i.e. additional to hypertension) of the metabolic syndrome (reduced HDL, raised triglycerides, glucose, waist circumference)*<br><br>* Definition of Metabolic Syndrome according to the International Diabetes Federation, 2006:<br>Central obesity (waist circumference > 94cm male (>90 if Asian), > 80 female<br>plus two of:<br>- SBP ≥ 130 or DBP ≥ 85 mmHg<br>- Fasting glucose >5.6mmol/l<br>- Fasting Triglycerides > 1.7 mmol/l (or on treatment)<br>- HDL < 1.03 mmol/l males, < 1.29 mmol/l females (or on treatment)

Supplementary Table 2

Exclusion criteria

1. Diabetes (types 1 or 2)
2. Secondary hypertension
3. eGFR < 45 mls/min
4. Plasma K^+ outside normal range on two successive measurements during screening
5. Clinic SBP >200 mmHg or DBP >120mmHg, with PI discretion to override if home BP measurements are lower
6. Requirement for diuretic therapy (other than for hypertension)
7. Absolute contra-indications to any of the study drugs (listed on their data-sheet)
8. Current therapy for cancer
9. Anticipation of change in medical status during course of trial (e.g. planned surgical intervention requiring >2 weeks convalescence, actual or planned pregnancy)
10. Inability to give informed consent
11. Not on stable doses of all hypertensive medications to be continued throughout the study for a minimum of 4 weeks prior to randomisation, or not normally less than 2 weeks if early randomisation is required at the discretion of the PI.
12. Participation in a clinical study involving an investigational drug or device within 4 weeks of screening.
13. Any concomitant condition that, in the opinion of the investigator, may adversely affect the safety and/or efficacy of the study drug or severely limit the subject's lifespan or ability to complete the study (eg, alcohol or drug abuse, disabling or terminal illness, mental disorders).
14. Treatment with any of the following prohibited medications:<br>a. Oral corticosteroids within 3 months of Screening.<br>b. Chronic use (defined as $\geq$3 days of treatment per week) of non-steroidal anti-inflammatory drugs (NSAIDs) other than acetylsalicylic acid.<br>c. The use of short-acting oral nitrates within 4 hours of screening or any subsequent study visit; long-acting oral nitrates (eg, Isordil) is permitted, but the dose must be stable for at least 2 weeks prior to screening and randomisation
15. A pill count will be made at the end of the 4 week run-in period and those with adherence <70% will be excluded from randomization

Supplementary Table 3
Footnotes to CONSORT diagram
(a) Reasons for non-randomisation

**Reasons for not randomising patients (not mutually exclusive)**	**N**
Patients not randomised	222
SBP too low	143
Withdrew consent	23
Not hypertensive by BHS criteria	21
Unspecified	17
No other component of metabolic syndrome	13
Diuretic not indicated	9
Adverse event	6
Adherence	6
Diabetes Type 1 or 2	4
Lost to follow up	3
Non-Compliance	2
Requirement for diuretic therapy other than for hypertension	2
Not on stable doses of all con meds for a min of 4 weeks prior to randomisation	2
Chronic stable or unstable use of NSAIDs	2
eGRF too low	2
Not aged 18-80	1
Participating in another study	1
Concomitant condition	1
Plasma K+ outside normal range on 2 successive measurements	1
SBP too high	1
Current therapy for cancer	1
Use of short-acting oral nitrates stable for 2 weeks prior to screening or any subsequent visit	1
Subject Unable To Swallow Medication	1
Patient Unable To Tolerate Oral Glucose Tolerance Test	1
Anticipation of change in medical status during course of trial (eg surgical intervention requiring GA, actual or planned pregnancy)	1

(b) Reason for non-inclusion in mITT population

Treatment Group	Reason for non-inclusion in mITT population	N
HCTZ	No primary outcome data	8
	Lost to follow up	1
	Investigator terminated participation	2
	Other Reason	1
	All	12
Amiloride	No primary outcome data	10
	Subject unwilling to continue	1
	Adverse Event	1
	Subject violated protocol	1
	All	13
	No primary outcome data	9
Combination	Subject unwilling to continue	2
	Adverse Event	3
	Investigator terminated participation	1
	Other Reason	2
	All	17

c. 'Other' reasons for discontinuation of randomised treatment

Treatment Group	Other reason
Amiloride	Low BP, cannot safely up-titrate medication
Combination	High K^+
	OGTT showed patient was diabetic
	Drug Hypersensitivity Reaction
	Ran out of tablets
HCTZ	Started oral corticosteroids, an exclusion
	Increased Glucose & HBA1c
	High OGTT results
	Personal reasons

Supplementary Table 4

Changes in 2-hour glucose – ITT and per protocol population

	Amiloride 10-20 mg	Amiloride 5-10 mg + HCTZ 12.5-25 mg	HCTZ 25- 50 mg
**ITT population (n=399)**	N=132	N=133	N=134
Absolute	6.68 (6.29,7.06)	6.90 (6.52,7.29)	7.40 (7.04,7.76)
Change from baseline	-0.52 (-0.91,-0.14)	-0.30 (-0.68,0.09)	+0.20 (-0.16,0.56)
Difference from HCTZ	0.72 (-1.20,0.25)	-0.50 (-0.98,0.025)	
	P=0.0026	P=0.039	
**Per protocol population (n=312)**	**N=92**	**N=92**	**N=105**
Absolute	6.73 (6.30,7.15)	6.85 (6.45,7.26)	7.44 (7.06,7.82)
Change from baseline	-0.38 (-0.80,0.04)	-0.25 (-0.66,0.15)	+0.33 (-0.05,0.71)
Difference from HCTZ	-0.71 (-1.21,-0.21)	-0.58 (-1.06,-0.08)	
	P=0.0054	P=0.021	

Least squares estimates adjusted for pre-specified baseline covariates in a mixed effects model, p values for comparisons with HCTZ.

Supplementary Table 4

Changes in 2 hour glucose from baseline to end of study, comparing HCTZ with the other two groups at high dose.

Supplementary Table 5

Blood pressure control rates

	**Amiloride**	**Combination**	**HCTZ**
	117*	121*	128*
Controlled (home SBP < 135 mmHg)	60	74	62
Unadjusted rates	51.3%	61.2%	48.4%
Least squares estimate (95% CI)	50.4 (41.4, 59.5)	61.7 (52.6, 70.0)	47.7 (39.0, 56.5)
Odds ratio (vs HCTZ)	1.12 (0.67, 1.86)	1.77 (1.05, 2.96)	
p-value (vs HCTZ)	0.67	0.031	

*mITT cohort using last recorded home BP

Supplementary Table 6

Predictors of changes in blood glucose and blood pressure

	Difference in 2 hour glucose at final visit	p value	Difference in home SBP at final visit	p value
Male vs Female	-0.80 (-1.36,-0.24)	0.0059	0.28 (-2.84,3.39)	
Age (per 10 years)	0.22 (-0.02,0.47)		-0.36 (-1.71,0.98)	
Waist (per 10 cm)	0.13 (-0.17,0.43)		0.66 (-0.70,2.02)	
BMI (per 10 units)	-0.06 (-0.50,0.38)		-0.31 (-2.76,2.15)	
Current smoker	-0.63 (-1.49,0.23)		0.44 (-4.07,4.94)	
Baseline HSBP (per mmHg)	-0.00 (-0.02,0.02)		0.44 (0.33,0.56)	<0.0001
Renin (per 10 fold difference)	-0.03 (-0.41,0.34)		4.75 (2.41,7.10)	<0.0001
Baseline K^+ (per mmol/L)	0.40 (-0.34,1.15)		-3.99 (-7.90,-0.09)	0.046
Baseline fasting insulin (per 100 units)	-0.16 (-0.74,0.43)		-3.04 (-6.56,0.47)	
Baseline 30 minute insulin (per pmol/L)	0.08 (-0.02,0.18)		0.51 (-0.08,1.11)	
Baseline fasting glucose (per mmol/L)	0.50 (0.12,0.87)	0.0096	0.78 (-1.03,2.59)	
Baseline 2 hr glucose (per mmol/L)	0.66 (0.54,0.77)	<0.0001	0.17 (-0.47,0.81)	
Baseline HbA1c (per %)	0.83 (0.12,1.54)	0.024	0.01 (-3.67,3.69)	

Least squares estimates from a multivariate model

Supplementary Table 7

All serious adverse events

Preferred term	Amiloride	Combination	HCTZ
Any	7	4	2
Abdominal pain	0	0	1
Atrial fibrillation	0	1	0
Back pain	1	0	0
Bile duct obstruction	0	1	0
Cellulitis	0	0	1
Chest pain	1	0	0
Diabetes mellitus	1	0	0
Forearm fracture	1	0	0
Haematemesis	1	0	0
Haematuria	0	0	1
Hospitalisation	1	0	0
Inguinal hernia repair	0	1	0
Joint surgery	1	0	0
Loss of consciousness	0	0	1
Myocardial infarction	0	1	0
Sepsis	0	1	0
Transurethral bladder resection	0	0	1
Vomiting	2	0	0

Distinct patients reporting serious adverse events with each preferred term. A single event may be coded with more than one preferred term.

Supplementary Table 8

Adverse events

	Amiloride (N=146)		Combination (N=150)		HCTZ (N=146)		
	n	%	n	%	n	%	p value
Dizziness	18	6.2	30	10.0	32	11.0	0.324
Muscle spasms	12	8.2	14	9.3	10	6.8	0.782
Headache	13	8.9	8	5.3	8	5.5	0.404
Nasopharyngitis	12	8.2	8	5.3	13	8.9	0.461
Diarrhoea	10	6.8	4	2.7	4	2.7	0.135
Lethargy	8	5.5	4	2.7	2	1.4	0.139
Arthralgia	4	2.7	8	5.3	6	4.1	0.549
Hyperkalaemia	7	4.8	3	2.0	0	0.0	0.017

Distinct patients reporting adverse events with each preferred term. Terms listed are those that occurred in at least 5% of patients on any treatment, or were significantly different between treatments ($p<0.05$). Exact P-values for Chi-Square tests.

Supplementary Table 8: Adverse events

Supplementary Figure 1

Schematic of the study

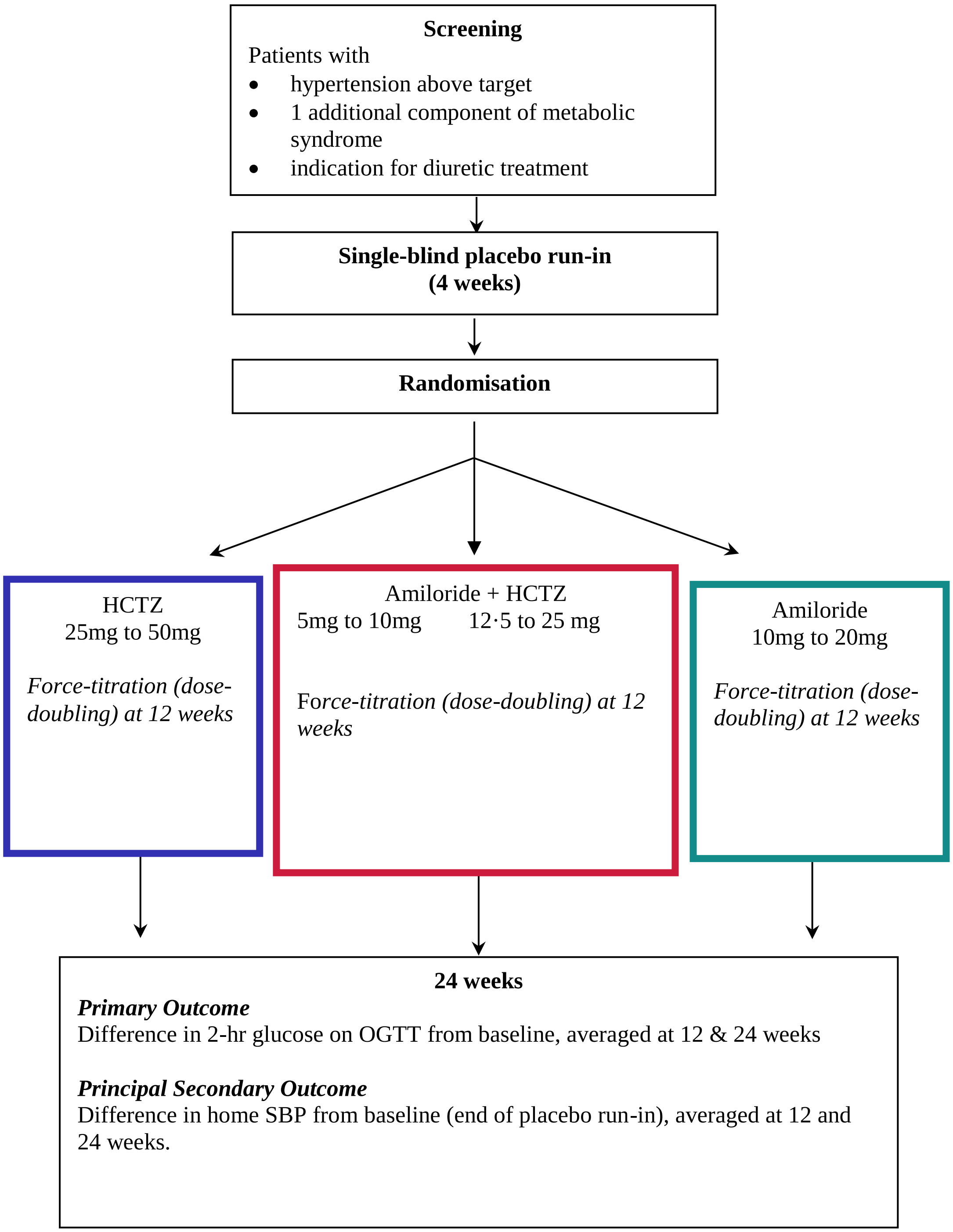

Supplementary Figure 2

Oral glucose tolerance tests

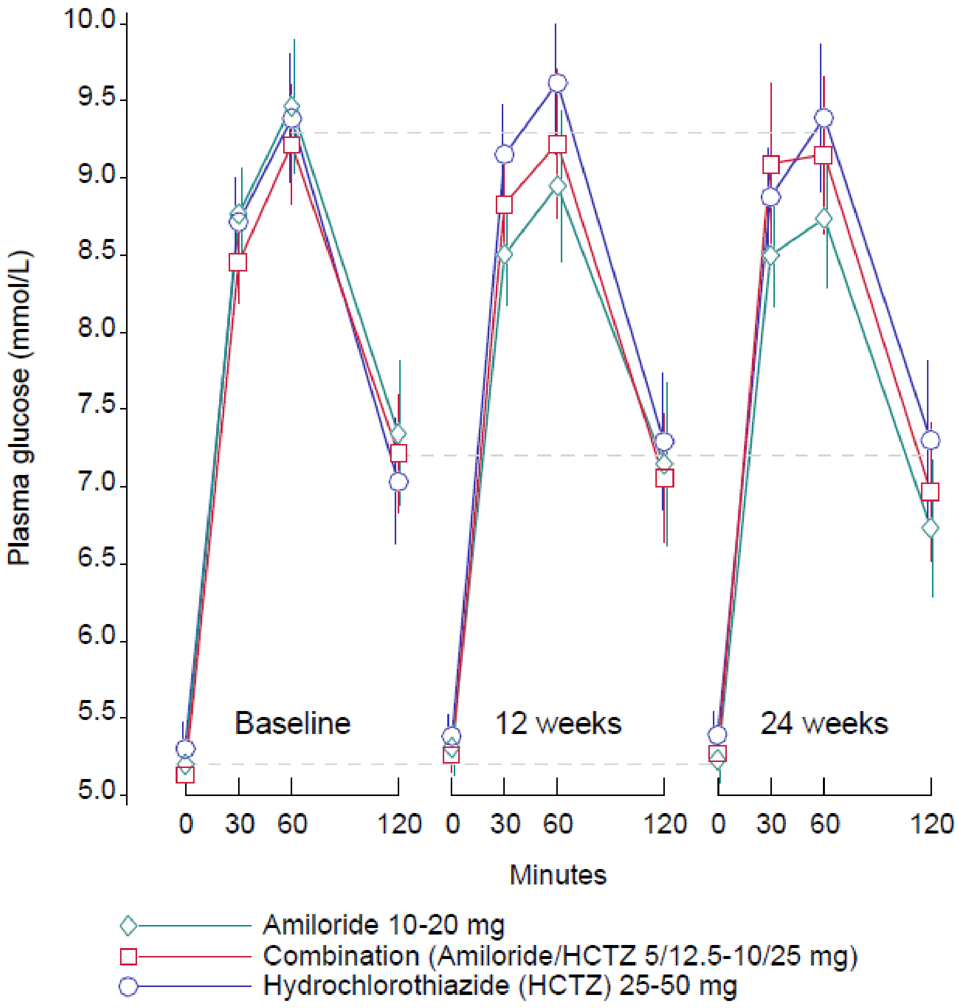

Unadjusted means (95% CI) for serum glucose during OGTT.
Area under the curve for glucose, averaged over 12 and 24 weeks, was 57.3 (95% CI 20.9,93.8) mmol*min lower on amiloride than HCTZ (p=0.0021). The difference between combination and HCTZ was 25.2 (-11.2,61.6) (p=0.17).

Supplementary Figure 3

Glucose vs potassium

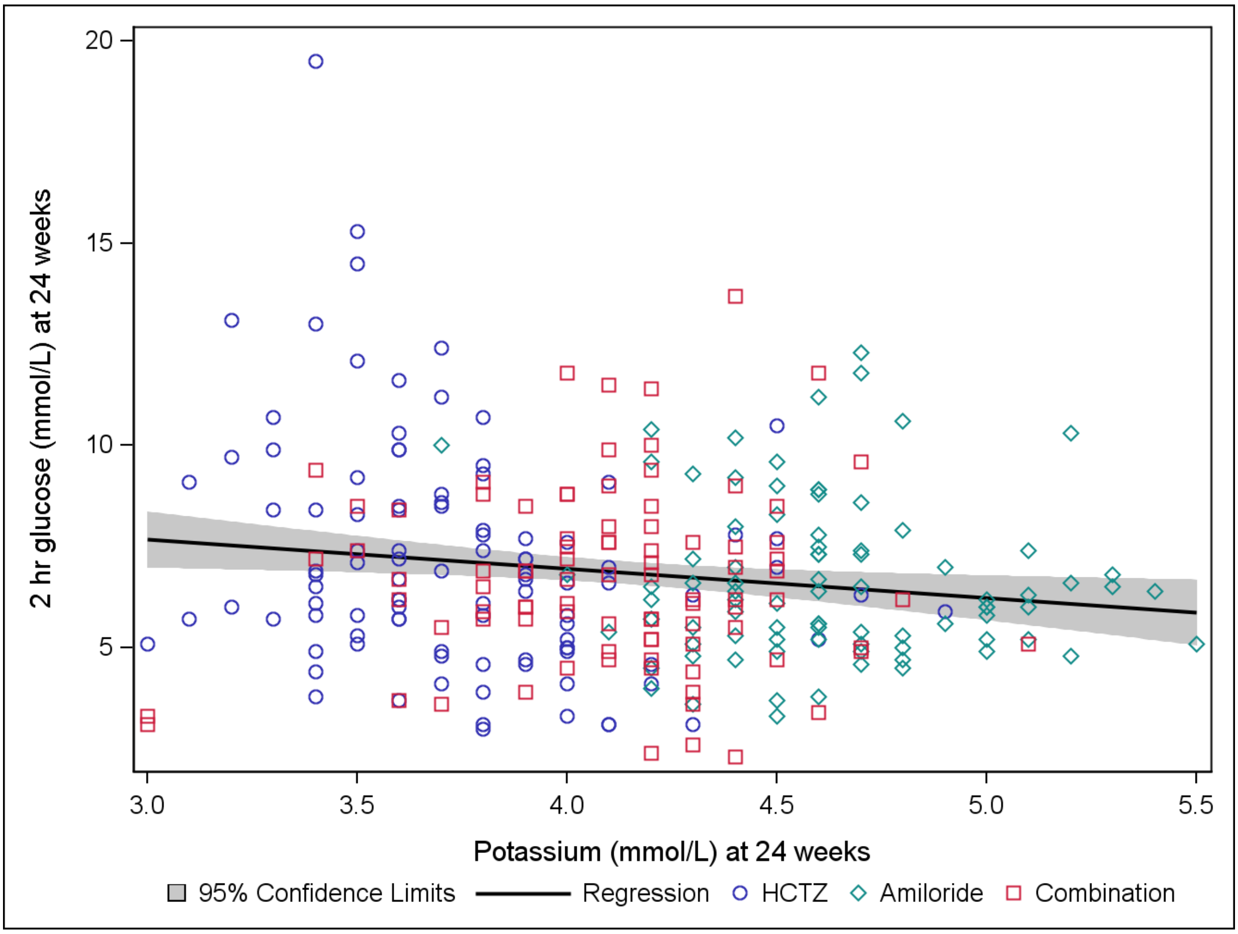

Regression of 24 hour glucose at 24 weeks on potassium concentrations at 24 weeks (N=314, r^2=0·02, p=0·010). No differences between treatments (p=0·60).

Supplementary Figure 4

Changes in electrolytes and eGFR

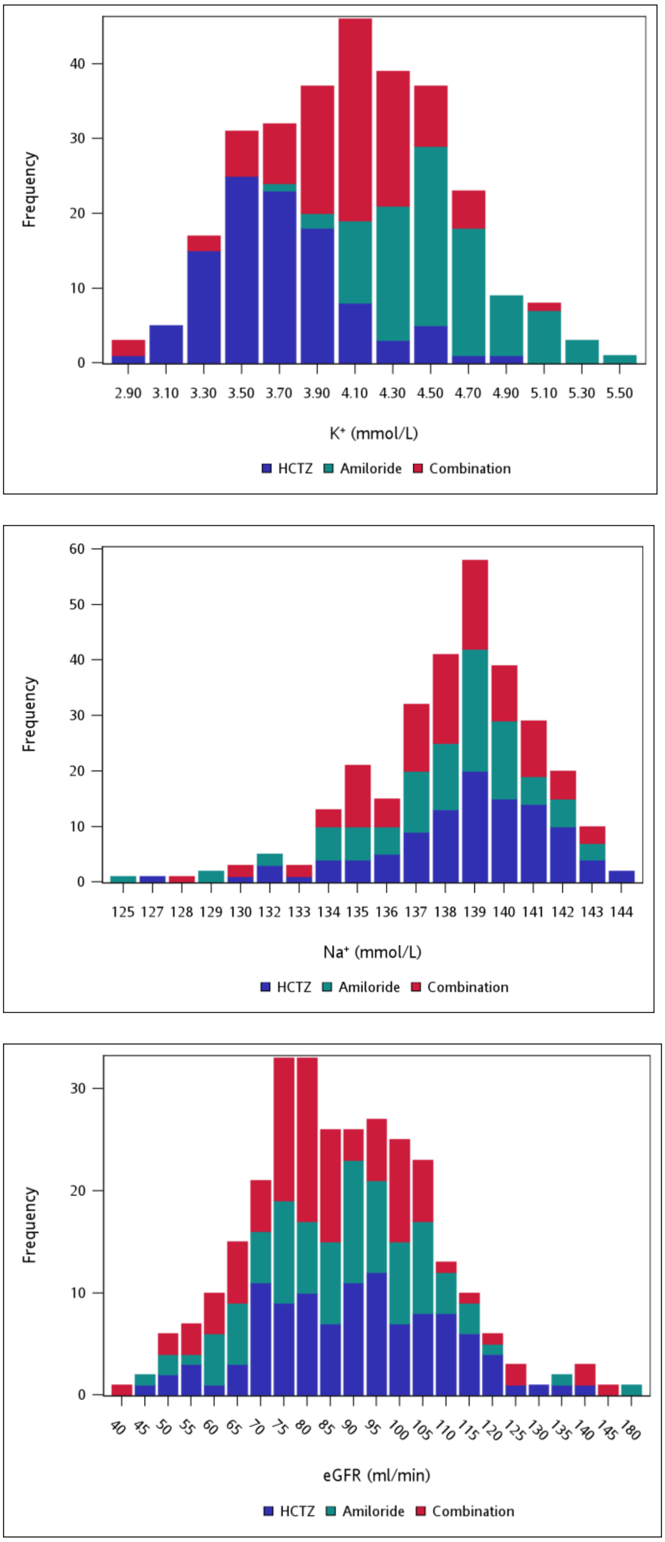

Results at final visit are shown as stacked histograms
